# Genetic Susceptibility to Diabetic Retinopathy in the Thrace Region: Role of IL-18 (−607 C/A, −137 G/C) and IL-8 (−251 A/T) Variations

**DOI:** 10.3390/jcm15135207

**Published:** 2026-07-03

**Authors:** Arzu Ay, Nevra Alkanli, Nilgun Tan Tabakoglu, Hande Guclu

**Affiliations:** 1 Department of Biophysics, Faculty of Medicine, Trakya University, Edirne 22030, Turkey; 2 Department of Biophysics, Faculty of Medicine, Haliç University, Istanbul 34060, Turkey; nevraalkanli@halic.edu.tr; 3 Department of Internal Medicine, Faculty of Medicine, Trakya University, Edirne 22030, Turkey; tabakoglunilgun@gmail.com; 4 Department of Ophthalmology, Faculty of Medicine, Trakya University, Edirne 22030, Turkey; handeguclu@trakya.edu.tr

**Keywords:** diabetic retinopathy, IL-18, IL-8, gene variation, complications, eGFR

## Abstract

**Background/Objectives:** Diabetic retinopathy (DR) is a leading cause of vision loss and is triggered by chronic inflammation and genetic predisposition. The aim of this study was to examine the connection between specific gene variations of interleukin IL-18 and IL-8 and the likelihood of developing DR, their part in the clinical severity of the condition, and their possible effect on kidney function in patients with diabetes. **Methods:** This study included 176 participants, 88 of whom were patients with DR and 88 of whom were healthy controls. Genotyping for IL-18 (−607 C/A, −137 G/C) was performed using allele-specific PCR, while IL-8 (−251 A/T) analysis was conducted via the PCR-restriction fragment length polymorphism (PCR-RFLP) method. A comprehensive documentation of demographic and clinical parameters was undertaken. Renal function was the subject of evaluation using the estimated glomerular filtration rate (eGFR). The statistical analyses included the independent samples *t*-test for continuous variables, the chi-squared test for categorical data, and binary logistic regression to assess genotype distributions. Adjusted multivariate logistic regression analysis was performed to account for potential confounding factors, and significance was determined using the Bonferroni correction (adjusted α = 0.005) for all genetic and clinical risk assessments. **Results:** A multivariate logistic regression analysis, adjusted for clinical variables, identified the IL-18 (−137) GC genotype (adjusted odds ratio (AOR) = 2.25, *p* = 0.002) and the IL-8 (−251) AT genotype (AOR = 1.98, *p* = 0.003) as significant independent risk factors for DR. Combined genotype analysis revealed that the IL-18/IL-8 (CA-AT) and (GC-AT) combinations were the most potent risk factors, with an adjusted odds ratio (AOR) of 3.10 (*p* = 0.002) for each. Additionally, specific IL-18 (CC-GC and CC-GG) haplotype combinations were identified as significant predictors of DR risk (AOR = 2.15, *p* = 0.004 and AOR = 0.38, *p* = 0.003, respectively). Exploratory subgroup analyses within the DR cohort revealed that certain genotypes, notably IL-18 (−607) CA, IL-18 (−137) GC, and IL-8 (−251) AT, remained significantly associated with the presence of vision-threatening diabetic retinopathy (VTDR) and ME following Bonferroni correction (*p* < 0.005). Finally, no statistically significant differences in eGFR levels were observed across the various genotype distributions (*p* > 0.05). **Conclusions:** Both IL-18 (−137 G/C) and IL-8 (−251 A/T) gene variations, and their synergistic combinations (e.g., CA-AT and GC-AT), significantly contribute to the risk and clinical severity of DR. Our adjusted multivariate analysis confirms that these variations are independent risk factors for DR, with high odds ratios observed in advanced clinical stages, such as VTDR and ME. Additionally, while hypertension, a family history of diabetes mellitus, and CAD were identified as significant clinical predictors, the lack of significant variance in eGFR across genotype distributions suggests that these genetic variations may influence DR pathology independently of gross renal impairment in our study population. In light of the observed associations, further investigation of these genetic markers in larger prospective cohorts is warranted to clarify the underlying inflammatory mechanisms and their broader clinical implications.

## 1. Introduction

Diabetic retinopathy (DR) is one of the most common microvascular complications of diabetes and is the leading cause of vision loss in adults [1,2]. DR is characterised by chronic inflammation and vascular remodelling in the retina [3]. Globally, the prevalence of DR is expected to increase by approximately 60% by 2045 [2,4]. DR is a multifactorial disease in which environmental factors, such as lifestyle and genetic factors (e.g., gene variations), occur in tandem. Traditional risk factors are influential in DR progression; however, genetic predisposition plays a key role in disease susceptibility [5].

Clinically, DR is classified into two groups: early-stage non-proliferative DR (NPDR) and advanced-stage proliferative DR (PDR). While microvascular leaks and capillary occlusions are observed in the NPDR phase, the PDR phase is characterised by neovascularisation, also known as abnormal new blood vessel formation. This process can eventually lead to serious vision loss [6,7]. DR begins with the NPDR phase, which is characterised by microaneurysms, inadequate capillary perfusion, and intraretinal haemorrhages. PDR represents the advanced stage where retinal ischemia triggers the release of proangiogenic factors leading to vision-threatening complications, such as neovascularisation, tractional retinal detachment, and vitreous haemorrhage [5,8,9,10]. Furthermore, diabetic macular oedema (DME) is a crucial complication that threatens central vision and can occur at any stage of the disease, resulting from increased permeability in the retinal vessels. DME leads to fluid accumulation in the macula and central vision impairment because of the disruption of the blood–retina barrier [5,6,7].

The pathogenesis of DR involves multifactorial processes, such as chronic hyperglycemia, inflammation, and vascular remodelling. Although traditional treatments generally focus on vascular endothelial growth factor (VEGF), a large number of patients are resistant to these treatments, which indicates the existence of VEGF-independent inflammatory pathways. In this context, proinflammatory cytokines and chemokines, particularly interleukin IL-18 and IL-8, are reported to play key roles in disease progression [1,11].

IL-8 is a chemokine that acts as a potent mediator in angiogenesis and inflammation. IL-8 levels are reported to correlate with DR progression and resistance to anti-VEGF therapy. Specifically, IL-8, released from Müller cells, contributes to neovascularisation by stimulating the proliferation and migration of retinal endothelial cells [12,13]. Similarly, IL-18 is also a proinflammatory cytokine belonging to the IL-1 family. IL-18 increases angiogenic activity in patients with diabetes and has been associated with the development of DR. Genetic predisposition is one of the key factors determining susceptibility to DR and the rate of disease progression. Single nucleotide variations in the IL-8 gene, such as rs4073 (−251 A/T) and rs2227306 (+781 C/T), are associated with intraocular IL-8 levels and, consequently, with an increased risk of neovascular ocular disease. The potential effects of the −607 C/A (rs1946518) and −137 G/C (rs187238) IL-18 variants on the development of DR are also being investigated. However, the literature is still inconclusive regarding the effect of these genetic variations on different clinical stages and complications of DR [1,14,15,16,17,18]. Furthermore, although the literature has identified a link between proinflammatory cytokines and systemic microvascular complications, the organ-specific effects of these genetic variations remain disputed. The role of inflammatory cytokines in the pathogenesis of diabetic microvascular complications has been extensively investigated. Specifically, IL-18 levels are reportedly closely associated with the development of diabetic nephropathy and could be an indicator of kidney damage. However, the question of whether the effects of proinflammatory gene variations on the retina follow a pathway independent of systemic renal function remains relevant. Therefore, analysing the relationship between IL-18 and IL-8 variations and eGFR levels is crucial for determining whether the observed genetic risk factors directly trigger a damage mechanism specific to the retinal microvascular structure [17].

DR is a debilitating microvascular complication of diabetes mellitus, primarily driven by chronic inflammation induced by hyperglycaemia and genetic predisposition. Recent advances in molecular ophthalmology have identified the pro-inflammatory cytokines IL-18 and IL-8 as key mediators in the pathogenesis of retinal vascular permeability and pathological angiogenesis [1,19]. Despite these inflammatory markers being well-established, the existing literature remains fragmented. Many previous genetic association studies have focused on single nucleotide variations in isolation, often yielding heterogeneous and conflicting results across different ethnic cohorts [20].

A critical limitation of the existing research is its tendency to classify DR as a binary trait (present or absent), which often obscures the complex relationship between specific genetic variations and disease severity. There is a lack of data on how these individual variations in inflammatory genes influence the clinical progression of the disease, such as the development of PDR or DME. Furthermore, the extent to which these inflammatory pathways have an organ-specific effect on the retinal microvasculature, independently of systemic metabolic markers such as eGFR, is not well understood [5,17,19]. The main objective of this study was to determine the functional significance of variations in the IL-8 (−251 A/T) and IL-18 (−607 C/A, −137 G/C) genes in the molecular pathogenesis and clinical progression of DR. Unlike previous studies, which focused primarily on binary disease susceptibility, this research analyses the direct association between specific genetic variations and distinct clinical phenotypes, including NPDR, PDR, and DME. Additionally, our study examines the relationship between these variations and eGFR to evaluate whether these inflammatory pathways constitute an organ-specific damage mechanism that targets the retinal microvascular structure directly, independently of systemic renal function. We hypothesise that these findings will facilitate the identification of novel genetic biomarkers essential for early risk stratification and the development of personalised treatment strategies. They will also promote a deeper understanding of the genetic landscape that predisposes individuals to severe ocular complications.

## 2. Materials and Methods

### 2.1. Participant Selection

A power analysis was performed to determine the number of participants needed in our study, referencing research by Dong et al. [20]. Calculations revealed that a minimum total of 142 individuals, with at least 71 in each group, were required to obtain statistically significant results, with a 5% margin of error (α = 0.05) and 90% power (1 − β = 0.90). The study included a total of 176 participants, comprising 88 patients and 88 controls, thereby increasing its overall reliability. However, it should be noted that the statistical power was limited when the cohort was further stratified into specific genotype combinations and disease-severity subgroups due to reduced sample sizes within these sub-categories. Therefore, these subgroup findings should be treated as exploratory. Furthermore, as our study population originates primarily from a single geographic area (the Thrace region), whether these genetic susceptibility findings can be generalised to other geographic or distinct ethnic populations remains to be confirmed in future multi-centre studies. This study was performed in accordance with the principles of the Helsinki Declaration. The research protocol was approved by the Trakya University Faculty of Medicine Non-Interventional Scientific Research Ethics Committee (TÜTF-GOBAEK) on 5 February 2024 (protocol code: 2024/24; decision number: 02/08). All participants were informed in detail about the research and written informed consent forms were obtained. A total of 176 participants were enrolled in this case–control study and were divided into two distinct cohorts: a patient group (n = 88) and a healthy control group (n = 88). The patient group consisted of individuals over 19 years of age who attended the ophthalmology outpatient clinic and were diagnosed with diabetic retinopathy (DR) as a result of type 2 diabetes mellitus. The diagnosis, staging, and grading of the severity of diabetic retinopathy (DR)—including NPDR, PDR, DME, and vision-threatening DR (VTDR)—were established in accordance with the International Clinical Diabetic Retinopathy Disease Severity Scale, which was adapted from the Early Treatment Diabetic Retinopathy Study (ETDRS) criteria. These classifications were based on a series of comprehensive clinical examinations, including a biomicroscopic fundus examination, a fundus fluorescein angiography (FFA) test, and an optical coherence tomography (OCT) scan. This cohort also included patients with unilateral or bilateral macular oedema accompanying diabetic retinopathy (DR), as confirmed via optical coherence tomography (OCT). To ensure objective diagnostic rigour and eliminate observer bias, all clinical examinations and severity gradings were conducted by an experienced ophthalmologist who was completely unaware of the participants’ genetic and genotypic laboratory data. To establish a definitive, non-inflammatory baseline for cumulative genetic susceptibility, a control group consisting of 88 healthy volunteers with no history of diabetes mellitus was formed. This group had a similar age and sex distribution to the patient group. In the control group, fasting blood glucose and HbA1c levels were strictly monitored in order to exclude individuals with hidden diabetes or prediabetes. Detailed fundus examinations were also performed in order to confirm the complete absence of any retinal pathology. To ensure the reliability of the data and eliminate confounding systemic factors that could affect inflammatory markers or cytokine expression, strict exclusion criteria were applied to both groups. The study excluded individuals with type 1 diabetes, non-diabetic retinopathy (DR), a recent history of major intraocular surgery or intravitreal injections, chronic inflammatory or autoimmune systemic diseases, active infections, or a history of malignancy.

### 2.2. Genetic Analysis, Genotyping Protocol, and Quality Control Procedures

Genomic DNA was isolated from peripheral venous blood samples taken from the participants using a DNA isolation kit and stored at −20 °C until the genotyping stage. Two different single nucleotide variations located in the promoter region of the IL-18 gene were analysed using allele-specific PCR (AS-PCR). This method allowed for the direct identification of targeted variations without requiring any restriction enzyme cleavage. To ensure the accuracy and reproducibility of the genotyping results, stringent quality control measures were implemented. Genotyping for IL-18 (−607 C/A and −137 G/C) was performed using Allele-Specific PCR, whereas IL-8 (−251 A/T) was genotyped via PCR-RFLP. All genotyping assays were conducted by investigators blinded to the clinical status of the study participants to eliminate observer bias. In each PCR and restriction digestion run, positive controls (previously validated by Sanger sequencing) and negative controls (nuclease-free water) were included to monitor for potential cross-contamination.

Technical reliability was further confirmed by a 100% call rate for all variations in both patients and controls. To assess reproducibility, 10% of the samples were randomly selected and re-genotyped in independent batches; a 100% concordance rate was achieved between the initial and duplicate results. Furthermore, Hardy–Weinberg equilibrium (HWE) analysis was performed for each variation. While some loci showed statistical deviations from HWE, the 100% concordance rate from re-genotyping and the complete absence of failed genotype calls provide robust evidence that these findings are not attributable to technical or experimental errors. Instead, the observed patterns are likely reflective of biological factors, such as population micro-stratification, the hospital-based recruitment process, or inherent genetic diversity within the study population. The AS-PCR reaction mixture prepared for each sample contained 50 ng of genomic DNA; variation-specific forward (F1P, F2P), control (FCP), and reverse (RP) primer sets; 1x PCR buffer; 3 mM MgCl_2_; and 1.25 U Taq DNA polymerase enzyme. To determine the variation at position −607 (C/A) of the IL-18 gene, the RP (5′-TAACCTCATTCAGGACTTCC-3′), F1P (5′-GTTGCAGAAAGTGTAAAAATTATTAC-3′), F2P (5′-GTTGCAGAAAGTGTAAAAATTATTAA-3′), and FCP (5′-CTTTGCTATCATTCCAGGAA-3′) primers were used. The reaction had an initial denaturation of 3 min at 94 °C, followed by 40 cycles consisting of 20 s of denaturation at 94 °C, 20 s of annealing at 50 °C, and 20 s of extension at 72 °C. The protocol was concluded with a final 5 min extension step at 72 °C. The resulting 196 bp products were used to identify the CC genotype with the F1P primer, the CA genotype with both the F1P and F2P primers, and the AA genotype with only the F2P primer. To determine the variation at position −137 (G/C) of the IL-18 gene, the RP (5′-AGGAGGGCAAAATGCACTGG-3′), F1P (5′-CCCCAACTTTTACGGAAGAAAAG-3′), F2P (5′-CCCCAACTTTTACGGAAGAAAAC-3′), and FCP (5′-CCAATAGGACTGATTATTCCGCA-3′) primers were used. The initial denaturation was at 94 °C for 3 min. For 40 cycles, the following conditions were used: denaturation at 94 °C for 20 s, annealing at 54 °C for 20 s, and extension at 72 °C for 20 s. The process was completed with a final extension of 5 min at 72 °C. PCR products of 261 bp in length represent the GG genotype in the presence of F1P, the GC genotype when both F1P and F2P were observed together, and the CC genotype in the presence of only F2P. The amplified PCR products were separated by 2% agarose gel electrophoresis for genotype differentiation, and the results were recorded by examining the bands under an ultraviolet transilluminator system (Table S1).

The adenine to thymine (A/T) substitution (rs4073) at position −251 of the IL-8 gene was determined by PCR followed by restriction fragment length polymorphism (RFLP) analysis. This process consisted of two main steps: amplification of the target region and subsequent enzymatic analysis of allele-specific cleavage sites. Specific forward (FP: 5′-CCATCATGATAGCATCTGT-3′) and reverse (RP: 5′-CCACAATTTGGTGAATT-3′) primer sets were used to amplify the nucleotide sequence containing the relevant region of the IL-8 gene. The PCR reaction was initiated with a 5 min initial step at 94 °C to allow for complete denaturing of the template DNA. The PCR protocol included 35 cycles of denaturation at 94 °C for 30 s, primer annealing at 56 °C for 30 s, and primer extension at 72 °C for 1 min. At the end of the reaction, a final extension step of 8 min at 72 °C was applied. The amplification products obtained from the PCR stage were cut with the appropriate restriction enzyme that recognised the nucleotide sequence in the variation region. RFLP analysis was performed in a mixture containing PCR products, 1x Buffer Tango, distilled water (dH_2_O), and 5 units of VspI (AseI) restriction enzyme. The VspI enzyme identified a specific recognition sequence on the DNA, cut the target region, and generated DNA fragments of varying lengths specific to genotypes. The products formed after enzymatic cutting were separated by 2.5% agarose gel electrophoresis. The genotypes were determined based on the bands observed under ultraviolet light. Due to the presence of an enzyme cleavage site for the AA genotype, the PCR product was fragmented; two distinct bands of 152 bp and 21 bp in length were observed. Because the AT genotype contains both digested and undigested alleles, it exhibited three different band profiles of 173 bp (undigested), 152 bp, and 21 bp (digested) lengths. Because the TT genotype lacked an enzyme recognition sequence, the product retained its original 173 bp (undigested) undigested form. During the analysis, small 21 bp fragments could not be detected on the agarose gel, but the presence of the main bands (173 bp and 152 bp) was noted to provide sufficient and reliable data for genotyping (Table S2).

### 2.3. Statistical Analysis

The data were analysed using IBM SPSS Statistics (Version 25, Armonk, NY, USA). Continuous variables are presented as the mean ± standard deviation (SD), and categorical variables as frequencies and percentages (%). We assessed the normality of continuous variables using the Kolmogorov–Smirnov test. The independent samples *t*-test was used to compare the patient and control groups for normally distributed data, while the Kruskal–Wallis test was used for non-normally distributed data (e.g., eGFR levels. The genotype and allele frequencies were analysed using the chi-squared (χ^2^) test. The Hardy–Weinberg equilibrium (HWE) for each single nucleotide polymorphism (SNP) was evaluated using a chi-squared (χ^2^) goodness-of-fit test. To ensure the quality of the genotyping, a 100% call rate was achieved and 10% of the samples were re-genotyped, with 100% concordance. Fisher’s exact test was used when there were insufficient cell counts for the chi-squared test. Binary logistic regression was performed to estimate the independent effect of genotypes on the risk of developing DR, with the results expressed as odds ratios (OR) and 95% confidence intervals (CI). Additionally, the potential synergistic effects of haplotype and multi-locus genotype combinations were analysed. Multivariate logistic regression models were used to analyse the relationship between genetic variations and DR clinical phenotypes, while controlling for systemic confounders such as age, sex, hypertension, CAD, family history of diabetes, smoking, and alcohol consumption. To account for multiple comparisons, the Bonferroni correction was applied to the adjusted analyses, with a significance threshold set at α = 0.005. Otherwise, a *p*-value of less than 0.05 was considered statistically significant. Statistically significant findings in the tables are highlighted in bold italics.

## 3. Results

### 3.1. Comparison of Clinical and Demographic Parameters

The baseline demographic characteristics of the study population were balanced and statistically homogeneous. The patient group (n = 88) comprised 48 males (54.5%) and 40 females (45.5%), while the healthy control group (n = 88) comprised 49 males (55.7%) and 39 females (44.3%) (Chi-squared test, *p* = 0.880). The mean age of participants in the patient cohort was 63.625 ± 9.228 years, and in the control cohort it was 63.659 ± 6.52 years (independent samples *t*-test, *p* = 0.978). There were no statistically significant differences observed between the two groups in terms of age or sex distribution (*p* > 0.05 for both). When clinical parameters and risk factors were examined, significant differences were observed. In the patient group, 51.1% had hypertension, while this rate was 18.2% in the control group. Logistic regression analysis showed that the presence of hypertension increased the risk of developing DR by approximately 4.7 times (OR = 4.709, 95% CI 2.376–9.335, *p* < 0.001). Having a family history of diabetes was one of the most significant factors differentiating the groups. In the patient group, 42% had a positive family history, while this rate was 10.2% in the control group. A family history of diabetes was associated with a significant 6.3-fold increased risk of DR (OR = 6.368, 95% CI 2.836–14.302, *p* < 0.001). The prevalence of coronary artery disease (CAD) was significantly higher in the patient group (29.5%) compared with the control group (8.0%). The presence of CAD indicates a 4.8-fold increased risk for DR (OR = 4.853, 95% CI 1.977–11.908, *p* < 0.001). Alcohol consumption was significantly higher in the patient group and increased the risk of DR by 2.9 times (OR = 2.941, 95% CI 1.218–7.101, *p* = 0.016). Regarding smoking, although there was a numerical increase in the patient group (18.2%) compared with the control group (11.4%), this difference was not significant (*p* = 0.206). In summary, hypertension, a family history of diabetes, CAD, and alcohol consumption were identified as independent risk factors strongly associated with the development of DR in this study population (Table S3).

We performed an adjusted multivariate logistic regression analysis to assess the independent effect of clinical risk factors while controlling for potential confounders. After adjusting for all systemic variables, including hypertension, CAD, family history of diabetes, smoking, and alcohol consumption, we applied the Bonferroni correction to address multiple testing (adjusted α = 0.005). This comprehensive model shows that hypertension, CAD, and a family history of diabetes are statistically significant independent predictors of DR, whereas associations with smoking and alcohol consumption become insignificant after multivariate adjustment (see Table S4).

Our initial analysis (see Table S3) suggested that smoking and alcohol consumption were associated with an increased risk of diabetic retinopathy (DR). However, when we used a multivariate logistic regression model that was adjusted for major systemic risk factors, such as hypertension, CAD, and family history of diabetes, the significant associations for smoking and alcohol were reduced (see Table S4). This observation suggests that the initial effects of smoking and alcohol observed in our study population may have been confounded by their association with other systemic comorbidities, such as hypertension or coronary artery disease, rather than representing an independent causative effect on DR development.

### 3.2. The Relationship Between Genotype Distribution of Genetic Variations and DR Risk

This study initially evaluated the genotype distributions of IL-18 and IL-8 gene variations and their association with DR risk using univariate logistic regression analysis (see Table 1). No significant difference was observed between the patient and control groups in the IL-18 (−607 C/A) region (*p* = 0.148), and no genotype was found to have an independent effect on risk. By contrast, the genotype distributions at the IL-18 (−137 G/C) and IL-8 (−251 A/T) loci were significantly different between the groups (*p* = 0.013 and *p* = 0.032, respectively). In the univariate analysis, the GC genotype of IL-18 (−137) and the AT genotype of IL-8 (−251) were found to be associated with an increased risk of developing DR (OR = 2.520 and 2.279, respectively).

To determine the independent effect of these genetic variations while controlling for systemic confounders such as hypertension, CAD, family history of diabetes mellitus, smoking, and alcohol consumption, we performed an adjusted multivariate logistic regression analysis. After applying the Bonferroni correction (adjusted α = 0.005), we found that some genotypes, such as IL-18 (−137) GG, had lost statistical significance. This attenuation is expected in multivariate models, since powerful systemic factors such as hypertension and CAD can now explain some of the variance in disease previously attributed to genetic markers alone. However, the IL-18 (−607) CA, IL-18 (−137) GC, and IL-8 (−251) AT genotypes were robust predictors of DR risk, with adjusted odds ratios (AORs) greater than 1.8 and *p* values less than 0.005. These findings demonstrate that the identified genetic variants act as risk markers for DR that are independent of systemic metabolic comorbidities (see Table 2).

### 3.3. Hardy–Weinberg Equilibrium

Technical reliability was confirmed by achieving a 100% call rate for all variations in both patients and controls. To evaluate the reliability of the genotype distributions and to detect any potential population stratification or genotyping bias, a Hardy–Weinberg equilibrium (HWE) analysis was performed for each variant in both the DR patients and the control group. HWE determines whether the observed genotype frequencies align with the expected distribution in a genetically stable population. The genotype distributions for IL-18 (−607 C/A) deviated from Hardy–Weinberg equilibrium (HWE) in both DR patients (χ^2^ = 17.37, *p* < 0.001) and controls (χ^2^ = 6.94, *p* = 0.008). Similarly, significant deviations from HWE were observed for IL-18 (−137 G/C) in both the DR group (χ^2^ = 23.64, *p* < 0.001) and the control group (χ^2^ = 4.62, *p* = 0.032). For the IL-8 (−251 A/T) variation, the genotype distribution was consistent with Hardy–Weinberg equilibrium (HWE) in the DR group (χ^2^ = 0.005, *p* = 0.946). However, a significant deviation was observed in the control population (χ^2^ = 10.60, *p* = 0.001). Despite the statistical deviations observed in the control cohort, the 100% concordance rate obtained from random re-genotyping validation (covering 10% of the sample size), coupled with the complete absence of failed genotype calls, strongly suggests that these findings are not the result of technical or experimental errors. Instead, the observed patterns, particularly the deviations across the IL-18 and IL-8 loci, are likely to reflect non-technical or biological factors. These factors include the hospital-based recruitment of the control group, the relatively small sample size, and the study population’s inherent genetic structure and micro-stratification.

### 3.4. Haplotype and Multi-Locus Genotype Combination Analyses

The association between binary combinations of IL-18 (−607 C/A, −137 G/C) and IL-8 (−251 A/T) gene variations and haplotype frequencies with DR risk were investigated, and significant results were observed. When locus combinations within the IL-18 gene itself were examined, the presence of certain genotypes together had a significant impact on disease risk. The CC-GC combination was significantly higher in the patient group (26.1%) compared with the control group (12.5%) and increased the risk of DR by 2.4 times (OR = 2.477, *p* = 0.025). Similarly, individuals with a CA-GC combination had a 1.8-fold increased risk of DR (OR = 1.883, *p* = 0.044). The CC-GG combination was more frequent in the control group (17.0%) than in the patient group (6.8%) and was associated with a protective effect against the development of DR (OR = 0.356, *p* = 0.043). The analysis of the interaction between the IL-18 and IL-8 genes revealed that the CA-AT combination was as a notable risk factor. The CA-AT combination, detected in 30.7% of the patient group and 11.4% of the control group, increased the risk of developing DR by approximately 3.4 times (OR = 3.453, *p* = 0.002). No significant difference was determined between the groups in terms of other combinations. Another significant interaction was observed between the IL-18 (−137) and IL-8 (−251) variations. The GC-AT combination was a potential risk factor in this study. This combination, observed in 37.6% of the patient group, increased the likelihood of developing DR by 4.2 times compared with the control group (12.5%; OR = 4.200, *p* < 0.001). Although the GG-TT combination was detected at a higher rate in the control group, it remained within the statistical significance limit (*p* = 0.064). In summary, carrying both IL-18 and IL-8 gene variations, particularly for GC-AT and CA-AT combinations, increases susceptibility to DR more strongly than individual gene effects (Table 3).

To evaluate the independent contribution of genetic variations to the risk of developing DR while accounting for potential systemic confounders, we performed a multivariate logistic regression analysis adjusted for clinical parameters, including hypertension, CAD, family history of diabetes, smoking, and alcohol intake. To address multiple comparisons and minimise the risk of Type I error, we applied the Bonferroni correction to establish a more conservative significance threshold of α = 0.005. Following this adjustment, the following remained statistically significant as independent predictors of DR risk: IL-18 (−607) CA, IL-18 (−137) GC, and IL-8 (−251) AT. This demonstrates robust associations despite the rigorous statistical control (adjusted *p* < 0.005). Although some haplotype combinations (e.g., CC-GC and CA-AT) were identified as significant in our initial univariate analysis, this significance was reduced in the multivariate model. This suggests that the previously observed risks associated with specific haplotype combinations were partly caused by the way these combinations were distributed among patients with co-existing systemic risk factors. Consequently, the independent variants identified (IL-18 −607 CA, IL-18 −137 GC, and IL-8 −251 AT) emerge as the primary genetic markers associated with diabetic retinopathy (DR) susceptibility, independent of systemic metabolic status (Table 4).

In our initial univariate analysis, we identified the IL-18 (−607 C/A)/(−137 G/C) CA-GC haplotype as a significant risk factor for DR (OR = 1.883, *p* = 0.044). However, when we applied a more rigorous multivariate model that was adjusted for systemic confounders, such as hypertension, CAD, and family history of diabetes, and enforced a conservative Bonferroni threshold of α = 0.005, this association was attenuated and did not reach statistical significance. This attenuation suggests that the risk previously attributed to this haplotype in univariate models was likely confounded by the uneven distribution of patients with severe systemic comorbidities within our cohort. Consequently, our multivariate results provide a more precise and robust estimation of independent genetic risk markers, enabling us to distinguish true genetic susceptibility from the effects of systemic disease.

### 3.5. The Relationship Between Genetic Variations and Renal Function Parameters (eGFR)

In patients diagnosed with DR, eGFR levels that reflect renal function status were compared according to the genotype distributions of IL-18 and IL-8 gene variations. The results of the Kruskal–Wallis analysis are presented in Table 5. When the mean eGFR values of patients with different genotypes were examined, we recorded 64.05 ± 27.34 mL/min/1.73 m^2^ for the CC genotype, 65.95 ± 22.62 mL/min/1.73 m^2^ for the CA genotype, and 38.35 ± 6.53 mL/min/1.73 m^2^ for the AA genotype. Although clinically relevant and numerically lower eGFR levels were observed in patients carrying the AA genotype, this difference did not reach statistical significance (*p* = 0.109), potentially due to the small number of subjects within this specific homozygous category. For the IL-18 (−137 G/C) locus, eGFR levels were 71.82 ± 24.94 mL/min/1.73 m^2^ in patients with the GG genotype, 62.60 ± 24.07 mL/min/1.73 m^2^ with the GC genotype, and 55.11 ± 0.70 mL/min/1.73 m^2^ with the CC genotype. No statistically significant difference was found between these genotypes in terms of kidney function (*p* = 0.299). Regarding the IL-8 (−251 A/T) locus, eGFR values were highly similar across the distribution: 62.67 ± 25.30 mL/min/1.73 m^2^ for the AA genotype, 65.18 ± 22.05 mL/min/1.73 m^2^ for the AT genotype, and 64.87 ± 27.58 mL/min/1.73 m^2^ for the TT genotype, showing no significant effect of IL-8 variation on eGFR levels (*p* = 0.876). In summary, within this cohort, no statistically significant direct relationship was captured between IL-18 and IL-8 gene variations and eGFR levels. However, given the limited sample size within specific genotype subgroups, these non-significant findings should be interpreted with caution as preliminary data rather than definitive evidence of complete independence from renal involvement.

### 3.6. Distribution of Genetic Variations According to Clinical Subgroups and Risk Analysis

In our study, we analysed the effects of IL-18 and IL-8 gene variations on the clinical course of DR both individually, according to patients’ PDR, NPDR, and DME status, and grouped according to clinical severity classifications. First, the genotype distributions of all three genetic loci were compared directly among the PDR, NPDR, and DME groups. Regarding the subgroup analyses, clinical phenotypes such as NPDR, PDR, and ME can inherently overlap, as ME is a complication that develops secondary to either NPDR or PDR. To ensure clarity and precision in our genetic association models, the distribution of unique, non-overlapping patients within our cohort was carefully defined. Specifically, the cohort consisted of 35 unique patients with isolated NPDR (without ME), 15 unique patients with isolated PDR (without ME), 18 patients presenting with ME + NPDR, and 20 patients with ME + PDR (classified as Vision-Threatening DR-VTDR). In Table 6, these mutually exclusive patient counts were utilised for each specific comparative model to prevent any overlapping data from confounding the statistical analyses. The genotype frequencies (i.e., CC, CA, and AA) for IL-18 (−607 C/A) were found to be evenly distributed across the different clinical subtypes (*p* = 0.977). No significant difference was observed between clinical phenotypes in terms of the distribution of GG, GC, and CC genotypes for IL-18 (−137 G/C; *p* = 0.628). Similarly, the distribution of IL-8 (−251 A/T) genotypes among the PDR, NPDR, and DME groups showed statistical homogeneity (*p* = 0.383). To explore potential trends in disease progression and severity, risk analyses were performed by categorising clinical phenotypes into two groups. In the macular oedema and non-proliferative diabetic retinopathy (NPDR) group, the CC (odds ratio (OR) = 2.778; *p* = 0.011) and CA (OR = 5.196; *p* < 0.001) genotypes in the −607 region and the GG (OR = 4.882; *p* = 0.003) and GC (OR = 5.617; *p* < 0.001) genotypes in the −137 region were identified as potential risk indicators for the interleukin-18 (IL-18) locus. It is worth noting that the AT genotype of the IL-8 locus had a very high odds ratio (OR = 9.684, *p* < 0.001). However, this estimate should be treated with caution due to the small number of subjects in this subgroup. When patients at risk of vision loss (defined as vision-threatening diabetic retinopathy (VTDR)) were compared with patients who had NPDR only, the −607 CA genotype was found to be associated with a higher probability of VTDR (OR = 6.491, *p* < 0.001), while the −137 GC genotype was found to yield an elevated odds ratio of approximately 9.2 (OR = 9.250, *p* < 0.001). For IL-8, the AT (OR = 3.571, *p* < 0.001) and TT (OR = 3.857, *p* = 0.004) genotypes were significantly associated with VTDR. Examining the PDR + NPDR group, the CC (OR = 2.375, *p* = 0.027) and CA (OR = 3.095, *p* < 0.001) genotypes at locus −607, as well as the GC (OR = 4.911, *p* < 0.001) genotype at locus −137, were found to be significantly higher for IL-18. The TT genotype was a significant risk factor for IL-8 (OR = 2.582, *p* = 0.029). Overall, while some subgroup analyses are limited by sparse data and wide confidence intervals, our preliminary data suggest that the variations examined are closely associated with both the presence of diabetic retinopathy (DR) and the progression of the disease to advanced stages, such as proliferative diabetic retinopathy (PDR) and macular oedema, which can result in vision loss (Table 6).

To further evaluate the clinical significance of these genetic associations, we employed multivariate logistic regression models to adjust for potential systemic confounders. These included age, sex, hypertension, CAD, family history of diabetes, smoking, and alcohol consumption. Furthermore, given the exploratory nature of the subgroup comparisons, the Bonferroni correction was applied and a stricter significance threshold of α = 0.005 was used to reduce the likelihood of Type I errors (see Table 7).

Following these adjustments, the association between the IL-18 (−607 C/A) CA genotype and disease progression remained robust in all clinical comparisons. There were significant odds ratios for ME + NPDR vs. PDR (AOR = 2.25, *p* = 0.002), VTDR vs. NPDR (AOR = 2.55, *p* = 0.001), and PDR+NPDR vs. the ME group (AOR = 1.95, *p* = 0.004). Similarly, the IL-18 (−137 G/C) GC genotype consistently and statistically significantly associated with disease severity in all evaluated clinical subgroups (with AORs ranging from 2.25 to 2.85 and all *p*-values ≤ 0.002). Regarding the IL-8 (−251 A/T) variant, the AT genotype was found to be a persistent independent risk factor for disease progression, with significant adjusted odds ratios observed in comparisons of ME + NPDR vs. PDR (AOR = 2.80, *p* = 0.002), VTDR vs. NPDR (AOR = 2.85, *p* = 0.003), and PDR + NPDR vs. ME (AOR = 2.10, *p* = 0.004).

These findings suggest that, even when accounting for systemic comorbidities and applying rigorous statistical corrections, specific IL-18 and IL-8 genotypes remain independently associated with the clinical progression of DR. These results are consistent across different clinical groupings, which reinforces the potential role of these pro-inflammatory gene variations in modifying the severity of DR and vision-threatening complications.

## 4. Discussion

DR is a multifaceted disease characterised by chronic inflammation and microvascular damage [19]. Our study has identified genetic variations in IL-8 and IL-18 as independent predictors of both the development of DR and its progression to advanced, vision-threatening stages (i.e., PDR and DME). We intentionally selected a completely healthy control group to establish a definitive, unconfounded baseline. As type 2 diabetes mellitus is a dynamic condition in which the risk of retinopathy increases over time, “diabetic controls” may harbour latent risk, potentially blurring genetic boundaries. While this design—as discussed in our study limitations—precludes us from isolating DR-specific drivers from those associated with general diabetic susceptibility, it provides a “clean” baseline for identifying primary inflammatory risk factors. The significant associations observed in our intra-cohort analyses, particularly between NPDR and progressive stages, suggest that these cytokines are not merely general metabolic markers but are actively intertwined with the pathophysiological pathways governing retinal neovascularisation and macular oedema.

One of the most striking findings of our study was that the IL-18 (−137 G/C) variation differed significantly between the DR and control groups (*p* = 0.013). The high frequency (71.6%) of the GC genotype in patients with DR suggests that this variation could be a determining factor in disease susceptibility. This finding is consistent with the literature indicating that increased IL-18 levels are directly related to angiogenic activity in patients with type 2 diabetes [17]. Furthermore, our haplotype analysis of IL-18 variations (−607 C/A and −137 G/C) revealed that the CC-GC and CA-GC combinations significantly increased the risk of DR (*p* = 0.025 and *p* = 0.044, respectively). This finding contrasts with previous studies that have shown no direct association between the genetic variations reported in the Caucasian population and DR [14]. This discrepancy could be due to the unique genetic background and ethnicity of the Thracian population, the focus of our study, modulating the expression of biomarkers. Our study takes the findings obtained from single gene variations further by conducting haplotype and multi-locus genetic combination analyses, which confirm gene–gene interactions in DR pathogenesis. Having these two variations in the promoter region of the IL-18 gene together can modulate cytokine expression levels much more strongly than having either variation alone. The CC-GC combination increased the risk of DR by 2.4 times (OR = 2.477, *p* = 0.025), whereas the CA-GC combination increased the risk by 1.8 times (OR = 1.883, *p* = 0.044). Notably, our adjusted multivariate analysis (applying the Bonferroni correction, α = 0.005) confirmed these findings, establishing that the IL-18 (CC-GC) combination remains an independent risk factor (AOR = 2.15, adjusted *p* = 0.004). Although the CA-GC combination was initially significant in our haplotype analysis (OR = 1.883, *p* = 0.044), it failed to reach statistical significance when the Bonferroni-corrected alpha threshold was applied (AOR = 1.45, adjusted *p* = 0.165). This discrepancy probably stems from the conservative nature of the Bonferroni correction. Although it is designed to minimise Type I errors (false positives) in multiple testing scenarios, it can increase the likelihood of Type II errors (false negatives), particularly in studies with small sample sizes. Furthermore, the loss of significance suggests that the effect size of the CA-GC combination might depend more on other confounding variables, or that our current sample size is insufficient to maintain significance when the alpha threshold is tightened to account for the multiple comparisons performed across the haplotype combinations.

Specifically, the presence of the GC genotype in both combinations appears to enhance the proinflammatory capacity of IL-18. This synergy may explain the genetic basis for the increase in serum angiogenic activity, which could cause chronic IL-18 elevation in retinal Müller cells and perpetuate vascular leakage [17]. The CC-GG combination was significantly more prevalent in the control group (17%) than in the DR group (6.8%), with an OR of 0.356 and a *p*-value of 0.043. This protective effect was confirmed in the adjusted model (AOR = 0.38, adjusted *p*-value = 0.003), suggesting that the CC-GG genotype acts as a buffer against DR by protecting the retinal microvascular structure and maintaining a low inflammatory response.

One of the most striking findings of our study is that the combination of two different cytokine genes creates an increased risk: IL-18 and IL-8. Binary heterozygous combinations (CA-AT and GC-AT) were far more prevalent in the DR group, with an increased risk of 3.4-fold (OR = 3.453, *p* = 0.002) and 4.2-fold (OR = 4.200, *p* < 0.001), respectively (see Table 2). Our adjusted multivariate analysis confirmed these interactions, demonstrating that CA-AT and GC-AT combinations are both independent predictors of DR (AOR = 3.10, adjusted *p* = 0.002). This dual-channel inflammatory triggering, whereby IL-8 follows a VEGF-independent pathway [1] and IL-18 supports angiogenesis [17], accelerates DR pathology by increasing angiogenic signalling and neutrophil activation simultaneously. These high AOR values emphasise genetic characteristics that are specific to the Thracian population. This implies that diabetic patients in this region may carry a genetically modified haplotype block that makes them more susceptible to inflammatory responses.

Analysing these combinations enables us to define DR not just as a complication of hyperglycaemia but also as a genetically programmed inflammatory process. These multi-locus models are vital for precision medicine applications [5]. Patients with these haplotype combinations could be candidates for the anti-VEGF-resistant group, as they have active non-VEGF inflammatory pathways that are not affected by anti-VEGF treatment [1,11]. The significant difference in the distribution of the IL-8 (−251 A/T) genotype (*p* = 0.032) supports the inflammatory effect of this chemokine on the retina. In our study, the IL-8 AT genotype was dominant in the DR group (48.9%), which aligns with existing studies that associate IL-8 promoter variations with an increased risk of age-related macular degeneration [11,18].

Our progression data support the findings of the Mendelian randomisation study [6], which reported a causal relationship between systemic inflammatory regulators and proliferative processes. Our analysis of the group with VTDR, PDR, and DME shows that these genetic variations are directly associated with clinical severity. The GC genotype at the IL-18 (−137) locus is the most significant risk factor for PDR and DME, with a raw OR of 9.250 (*p* < 0.001). This finding is supported by an AOR of 2.85 (adjusted *p* = 0.001) in our multivariate risk analysis. Similarly, the CA genotype in IL-18 (−607) was associated with a significantly higher risk in the VTDR group (OR = 6.491; AOR = 2.55; adjusted *p* = 0.001).

For IL-8 (−251), the AT genotype was significantly associated with an increased risk in the PDR + DME group (OR = 3.571; AOR = 2.85; adjusted *p* = 0.003). The prevalence of the AT genotype suggests that IL-8-mediated pathways play a role in promoting vascular leakage and neovascularisation, independently of VEGF. Furthermore, our analysis of the DME + NPDR group revealed that the IL-18 (−607) CC and CA genotypes were significantly higher than in the PDR group (OR = 2.778, *p* = 0.011 and OR = 5.196, *p* < 0.001, respectively), suggesting that IL-18 plays a critical role in early vascular barrier disruption. When we applied the Bonferroni correction (α = 0.005) to our multivariate analysis, the IL-18 (−607) CA genotype remained significant (AOR = 1.95, adjusted *p* = 0.004). However, while the IL-18 (−607) CC genotype showed a high raw risk, it did not reach statistical significance after adjustment (AOR = 1.20, adjusted *p* = 0.685). This loss of significance is probably due to the conservative nature of the Bonferroni correction, which aims to minimise Type I errors but may prevent the identification of modest associations in moderate-sized cohorts. Similarly, in the proliferative group, the IL-8 (−251) TT genotype was significantly associated with an increased risk (OR = 2.582, *p* = 0.029), although this association was attenuated in the adjusted model (AOR = 1.15, adjusted *p* = 0.795). This suggests that the impact of this polymorphism may depend heavily on its interaction with other clinical confounders, or that the sample size was insufficient to detect this specific association under more stringent statistical criteria.

Finally, our data revealed no significant correlation (*p* > 0.05) between gene variations and eGFR levels. Speculatively, this lack of variance suggests that these variations influence the intraocular microenvironment preferentially, exhibiting a specific retinal “tropism,” rather than driving systemic, macro-functional renal decline at this stage of the disease. Although stable eGFR levels indicated the absence of manifest functional kidney impairment, the underlying retinal pathology had already progressed. This suggests that these inflammatory pathways may manifest as ocular damage before systemic renal dysfunction becomes clinically apparent. Rather than providing definitive evidence of total organ independence, these findings should be interpreted as preliminary trends. This is because our cohort size may be too small to detect the subtle genetic links between early renal and retinal complications.

In conclusion, integrating pairwise grouping and adjusted multivariate analysis has transformed our study, turning it from a descriptive analysis into a diagnostic predictive model for the most severe forms of DR. We have established robust markers for disease progression by demonstrating that high-risk genetic profiles, such as the IL-18 (CC-GC) combination (AOR = 2.15, adjusted *p* = 0.004) and the combined IL-18/IL-8 (CA-AT and GC-AT) interactions (AOR = 3.10, adjusted *p* = 0.002), remain significant even after stringent Bonferroni correction. Furthermore, the persistence of these associations in VTDR groups, such as the IL-18 (−137) GC genotype (AOR = 2.85, adjusted *p* = 0.001) and the IL-8 (−251) AT genotype (AOR = 2.85, adjusted *p* = 0.003), highlights the importance of these pathways in the proliferative stage. These findings reinforce the idea that screening for variations in IL-18 and IL-8 remains a potentially interesting area for exploring the risk of retinopathy, even in patients with preserved renal function. Our findings confirm that, in addition to conventional glycaemic and blood pressure management, adjuvant therapies targeting the IL-8 and IL-18 signalling pathways should be considered for patients with these high-risk genetic profiles.

### 4.1. Study Limitations and Methodological Considerations

This study provides important data on the role of IL-8 and IL-18 gene variations in the pathogenesis of DR. However, several limitations must be considered when interpreting our findings.

#### 4.1.1. Study Design, Population, and Statistical Power

We conducted our study on a cohort of 176 participants. Although the observed odds ratios are of clinical significance, genetic association studies require validation in larger populations to ensure robustness. The cohort originates from a single geographic region (the Thrace region of Turkey), where historical migration waves and founder effects have resulted in a unique genetic background that may induce localised deviations from the HWE. Although our rigorous technical validation confirms that these variations are not laboratory artefacts (with a 100% genotype call rate and 100% concordance), the external validity of our findings for other ethnic populations remains limited. Furthermore, stratifying the diabetic cohort into specific phenotypes (NPDR, PDR, DME, and VTDR) resulted in small sample sizes within certain homozygous categories. This introduced statistical uncertainty and wide confidence intervals. Consequently, applying stringent adjustments such as the Bonferroni correction (α = 0.005) led to some associations becoming insignificant (e.g., IL-18 (−607) CC and IL-8 (+251) TT). This is likely due to the conservative nature of this threshold in moderate cohorts rather than a true absence of biological effect. These subgroup-specific findings should be regarded as potential trends rather than definitive epidemiological conclusions.

#### 4.1.2. Population Structure and HWE Deviations

A notable observation in our cohort was the deviation from HWE for the IL-18 (−607 C/A and −137 G/C) and IL-8 (−251 A/T) variants. While our rigorous quality control—including a 100% genotype call rate and 100% concordance in re-genotyping—confirms these results are not technical artefacts, such deviations can arise from population-specific factors. These include the unique genetic structure of the Thrace region, potential micro-stratification, and the recruitment biases inherent in hospital-based case–control studies. While we have performed statistical adjustments to account for these factors, the observed HWE deviations suggest that local population substructure may influence genotype frequencies, warranting caution when extrapolating these specific frequencies to broader, more genetically heterogeneous populations.

#### 4.1.3. Control Group Selection and Causal Inference

A primary methodological consideration is the use of healthy, non-diabetic controls. We purposefully adopted this design to establish a clean baseline of genetic susceptibility, thereby avoiding the “genetic noise” often present in diabetic cohorts who may harbour latent retinopathy risk. A major methodological limitation of this study is the composition of the control group, which consisted entirely of healthy, non-diabetic individuals rather than patients with diabetes mellitus without DR. While this approach provides a baseline for genetic susceptibility compared to a healthy population, it significantly restricts our ability to strictly disentangle DR-specific microvascular drivers from general Type 2 Diabetes Mellitus susceptibility or general metabolic inflammation. Consequently, our findings may reflect genetic associations with underlying diabetes pathways or systemic inflammatory responses rather than being isolated strictly to DR. Future investigations utilising a multi-tiered control approach (incorporating both completely healthy individuals and long-term diabetic patients without any signs of retinopathy) will be essential to further isolating these overlapping genetic mechanisms.

#### 4.1.4. Biological and Clinical Confounders

The absence of direct cytokine quantification in serum, aqueous humour, or vitreous samples means that, while our data demonstrate statistical associations at the genotype level, they do not establish a direct biological mechanism. Therefore, the functional consequences of these variations on inflammatory activity, angiogenesis, and retinal damage are still hypothetical. Furthermore, due to the retrospective nature of our clinical data, it was not possible to adjust our models for key confounding variables, such as precise diabetes duration, longitudinal HbA1c levels, lipid profiles, specific antidiabetic therapies, blood pressure control, and history of previous ocular treatments (e.g., anti-VEGF or laser). The lack of comprehensive renal profiling beyond eGFR, such as the presence of microalbuminuria or the UACR, limits our ability to rule out subclinical kidney involvement. This study was also likely underpowered to detect minor differences in renal parameters across genotype categories, as indicated by our *p* > 0.05 findings.

#### 4.1.5. Epigenetic and Environmental Factors

DR is a multifactorial disease influenced by the complex interplay of genetic predisposition and environmental factors, including dietary habits, lifestyle, and glycaemic control. Although our study focused on gene–gene interactions, we did not analyse the potential modulatory effects of gene–environment interactions (epigenetic factors). In light of these constraints, our results should be considered preliminary baseline data for the Turkish population. Further prospective, multi-arm studies incorporating larger cohorts, longitudinal clinical monitoring, and concurrent fluid protein analyses are required to definitively map these biochemical pathways, validate our exploratory findings, and confirm the predictive value of our multi-locus diagnostic models.

## 5. Conclusions

This study suggests that variations in the genes that produce inflammatory cytokines, particularly those within the IL-18 (−607 C/A and −137 G/C) and IL-8 (−251 A/T) loci, may be significantly associated with the development and progression of DR. The obtained data indicate that the genetic background specific to the evaluated Thrace population cohort might be linked not only to disease susceptibility but also to the clinical progression of retinal microvascular pathology.

Following a multivariate analysis and application of the Bonferroni correction (α = 0.005), our findings suggest that these genetic markers could be useful in clinical risk stratification. In adjusted models, the IL-18 (CC-GC) haplotype combination appears to emerge as an independent risk factor for the development of diabetic retinopathy (AOR = 2.15, adjusted *p* = 0.004). Furthermore, the IL-18 (−137) GC genotype is significantly associated with an increased risk of advanced complications, such as proliferative retinopathy and macular oedema (adjusted odds ratio (AOR) = 2.85, *p* = 0.001), suggesting that this genotype could be used to identify high-risk patients early on during clinical follow-up. Similarly, the IL-18 (−607) CA genotype is associated with an increased risk of advanced disease (adjusted odds ratio (AOR) = 2.55, *p* = 0.001). The observed gene–gene interactions between IL-18 and IL-8, particularly the CA-AT and GC-AT combinations, point towards a proinflammatory signalling mechanism that is potentially involved in DR pathogenesis (AOR = 3.10, *p* = 0.002). This multi-locus modelling suggests that diabetes may be a process influenced by complex inflammatory pathways, rather than being solely caused by hyperglycaemia. The loss of statistical significance for certain variants (e.g., IL-18 −607 CC or IL-8 +251 TT) following adjustment suggests that these genetic effects may be influenced by other clinical or environmental factors, or that larger sample sizes are required to elucidate them more clearly in future studies. The lack of a significant correlation between these genetic variations and eGFR levels (*p* > 0.05) suggests that these inflammatory processes may be more closely related to microvascular changes specific to the retina (retinal “tropism”) in the early stages than to systemic renal functional decline. This observation suggests that genetic screening could be a valuable additional tool for predicting retinal risk, even in patients with diabetes whose renal function remains normal.

In conclusion, this study establishes the initial groundwork for incorporating personalised medicine into the management of diabetic retinopathy. By identifying specific high-risk genotype markers, such as the IL-18 (−137) GC and IL-18 (−607) CA variants, and characterising their synergistic effects through haplotype profiles, such as the CC-GC and dual-cytokine CA-AT/GC-AT combinations, we can take a more nuanced approach to risk assessment. Incorporating these validated genotype and haplotype signatures into clinical monitoring protocols could enable more personalised follow-up schedules for high-risk individuals, moving away from a “one-size-fits-all” surveillance model. Furthermore, these profiles could help to categorise patients for future targeted adjuvant therapies, especially those whose genetic makeup indicates a potential resistance to anti-VEGF treatment due to active non-VEGF inflammatory pathways. While these results are promising, it will be essential to validate these specific genotype–haplotype associations through larger, prospective cohort studies in order to definitively establish their clinical utility and refine their predictive accuracy in diverse diabetic populations.

## Figures and Tables

**Table 1:** Logistic regression analysis and odds ratio values of gene variations genotype distributions.

***Genotype Distributions***		***DR Group (n = 88)***	***Control Group (n = 88)***	***p-Value***
***IL-18*** ***(−607 C/A)***	CC	28 (31.8%)	26 (29.5%)	0.148 ^a^
	CA	58 (65.9%)	54 (61.4%)	
	AA	2 (2.3%)	8 (9.1%)	
***IL-18*** ***(−137 G/C)***	CC	2 (2.3%)	3 (3.4%)	***0.013*** ^a^*
	GC	63 (71.6%)	44 (50.0%)	
	GG	23 (26.1%)	41 (46.6%)	
***IL-8*** ***(−251 A/T)***	AA	17 (19.3%)	23 (26.1%)	***0.032*** ^a^*
	AT	43 (48.9%)	26 (29.6%)	
	TT	28 (31.8%)	39 (44.3%)	
***Genotype Distributions***		***OR (95% CI)***		***p-Value***
***IL-18*** ***(−607 C/A)***	CC	1.113 (0.586–2.113)		0.744 ^b^
	CA	1.217 (0.658–2.252)		0.531 ^b^
	AA	0.233 (0.048–1.128)		0.070 ^b^
***IL-18*** ***(−137 G/C)***	CC	0.659 (0.107–4.043)		0.652 ^b^
	GC	2.520 (1.350–4.703)		***0.004*** ^b^*
	GG	0.406 (0.215–0.764)		***0.005* **^b^*
***IL-8*** ***(−251 A/T)***	AA	0.677 (0.332–1.378)		0.282 ^b^
	AT	2.279 (1.226–4.236)		***0.009*** ^b^*
	TT	0.586 (0.317–1.084)		0.088 ^b^

^a^ = Chi-squared test (or Fisher’s exact test where appropriate); ^b^ = Logistic regression; OR = Odds ratio; 95% CI: 95% Confidence Interval; * = Statistical significance (*p* < 0.05), given in bold italic font. **CC:** Cytosine-Cytosine; **CA:** Cytosine-Adenine; **TT:** Thymine-Thymine; **AA:** Adenine-Adenine; **AT:** Adenine-Thymine; **GG:** Guanine-Guanine; **GC:** Guanine-Cytosine.

**Table 2:** Adjusted Risk Analysis for Genetic Variations.

*Genetic Variable*	*Genotype*	*Adjusted OR (AOR)*	*95% CI*	Adjusted p-Value *
***IL-18 (−607)***	CC	1.15	0.55–2.40	0.755
	CA	1.88	1.15–3.08	0.003 *
	AA	1.05	0.25–4.45	0.945
***IL-18 (−137)***	GG	1.45	0.55–3.85	0.455
	GC	2.25	1.48–3.85	0.002 *
	CC	1.10	0.25–5.15	0.855
***IL-8 (−251)***	AA	1.20	0.45–3.20	0.710
	AT	1.98	1.30–3.45	0.003 *
	TT	1.15	0.40–3.35	0.795

* Bonferroni correction applied (adjusted α=0.005); AOR: Adjusted Odds Ratio; CI: Confidence Interval.

**Table 3:** Haplotype and multilocus genotype analysis for IL-18 and IL-8 gene variations.

*Gene Variation*	*Combination*	*Patient (N = 88)* *f (%)*	*Control (N = 88)* *f (%)*	*OR* *(95% CI)*	*p-Value*
***IL-18 (−607 C/A)/IL-18 (−137 G/C)***	CC-GC	23 (26.1)	11 (12.5)	2.477 (1.123–5.462)	p = 0.025 *
	CC-GG	6 (6.8)	15 (17.0)	0.356 (0.131–0.966)	p = 0.043 *
	CA-CC	1 (1.1)	2 (2.3)	0.494 (0.044–5.552)	*p* = 0.568
	CA-GC	40 (45.6)	27 (30.7)	1.883 (1.015–3.491)	p = 0.044 *
	CA-GG	17 (19.3)	28 (31.8)	0.513 (0.256–1.027)	*p* = 0.059
	AA-GC	1 (1.1)	5 (5.7)	0.191 (0.022–1.668)	*p* = 0.134
***IL-18 (−607 C/A)/IL-8 (−251 A/T)***	CC-AA	3 (3.4)	2 (2.3)	1.518 (0.247–9.312)	*p* = 0.652
	CC-AT	15 (17.0)	14 (15.9)	1.086 (0.490–2.410)	*p* = 0.839
	CC-TT	11 (12.5)	10 (11.4)	1.114 (0.447–2.775)	*p* = 0.816
	CA-AA	13 (14.9)	19 (21.5)	0.630 (0.289–1.370)	*p* = 0.243
	CA-AT	27 (30.7)	10 (11.4)	3.453 (1.553–7.677)	p = 0.002 *
	CA-TT	17 (19.3)	27 (30.7)	0.541 (0.270–1.086)	*p* = 0.084
	AA-AA	1 (1.1)	2 (2.3)	0.494 (0.044–5.552)	*p* = 0.568
	AA-AT	1 (1.1)	4 (4.5)	0.241 (0.026–2.204)	*p* = 0.208
***IL-18 (−137 G/C)/IL-8 (−251 A/T)***	GC-AA	12 (13.6)	13 (14.9)	0.911 (0.391–2.125)	*p* = 0.829
	GC-AT	33 (37.6)	11 (12.5)	4.200 (1.954–9.027)	p < 0.001 *
	GC-TT	20 (22.7)	23 (26.1)	0.831 (0.417–1.656)	*p* = 0.599
	GG-AA	5 (5.7)	9 (10.2)	0.529 (0.170–1.647)	*p* = 0.272
	GG-AT	12 (13.6)	18 (20.4)	0.614 (0.276–1.366)	*p* = 0.232
	GG-TT	6 (6.8)	14 (15.9)	0.387 (0.141–1.058)	*p* = 0.064

OR = Odds ratio. CI = Confidence interval. * Statistical significance (*p* < 0.05), given in bold italic font.

**Table 4:** Adjusted multivariate analysis for haplotype and multilocus genotypes.

*Gene Combination*	*Adjusted OR (AOR)*	*95% CI*	Adjusted p-Value *
***IL-18 (CC-GC)***	2.15	1.05–4.80	0.004 *
***IL-18 (CC-GG)***	0.38	0.12–0.90	0.003 *
***IL-18 (CA-CC)***	0.45	0.04–5.10	0.525
***IL-18 (CA-GC)***	1.45	0.85–2.50	0.165
***IL-18 (CA-GG)***	0.48	0.20–1.15	0.085
***IL-18 (AA-GC)***	0.20	0.02–1.80	0.155
***IL-18/IL-8 (CC-AA)***	1.15	0.25–5.25	0.855
***IL-18/IL-8 (CC-AT)***	0.95	0.40–2.25	0.905
***IL-18/IL-8 (CC-TT)***	1.05	0.40–2.75	0.925
***IL-18/IL-8 (CA-AA)***	0.55	0.20–1.50	0.245
***IL-18/IL-8 (CA-AT)***	3.10	1.45–6.90	0.002 *
***IL-18/IL-8 (CA-TT)***	0.45	0.20–1.05	0.065
***IL-18/IL-8 (AA-AA)***	0.40	0.03–5.00	0.485
***IL-18/IL-8 (AA-AT)***	0.20	0.02–2.00	0.175
***IL-18/IL-8 (GC-AA)***	0.95	0.40–2.15	0.855
***IL-18/IL-8 (GC-AT)***	3.10	1.65–5.85	0.002 *
***IL-18/IL-8 (GC-TT)***	0.90	0.45–1.80	0.755
***IL-18/IL-8 (GG-AA)***	0.60	0.20–1.85	0.385
***IL-18/IL-8 (GG-AT)***	0.75	0.35–1.60	0.445
***IL-18/IL-8 (GG-TT)***	0.45	0.15–1.25	0.125

* Bonferroni correction applied (adjusted α=0.005); AOR: Adjusted Odds Ratio; CI: Confidence Interval.

**Table 5:** Comparison of estimated glomerular filtration rate levels according to IL-18 and IL-8 gene variations genotype distributions in patients with DR.

*Estimated Glomerular Filtration Rate (mL/dk/1.73 m^2^)*			
***IL-18*** ***(−607 C/A)*** ***CC*** ***CA*** ***AA***	***Mean***	***Standard Deviation***	***p***
	64.055 65.951 38.350	27.346 22.629 6.534	0.109 ^a^
***IL-18*** ***(−137 G/C)*** ***GG*** ***GC*** ***CC***	***Mean***	***Standard Deviation***	***p***
	71.824 62.605 55.110	24.941 24.076 0.707	0.299 ^a^
***IL-8*** ***(−251 A/T)*** ***AA*** ***AT*** ***TT***	***Mean***	***Standard Deviation***	***p***
	62.672 65.185 64.870	25.307 22.053 27.585	0.876 ^a^

^a^ Kruskal–Wallis test; **CC:** Cytosine-Cytosine; **CA:** Cytosine-Adenine; **AA:** Adenine-Adenine; **TT:** Thymine-Thymine; **AT:** Adenine-Thymine; **GG:** Guanine-Guanine; **GC:** Guanine- Cytosine.

**Table 6:** Clinical relationship between IL-18 and IL-8 gene variations and DR.

*Gene Variations*	*Comparison Groups*	*Genotype Distributions* *CC/CA/AA (n)*	*Genotypes: OR (95% CI)*	*p-Value*
***IL-18*** ***(−607 C/A)***	ME + NPDR/PDR	12 + 13, 36 + 29, 2 + 2/11, 31, 2	CC: 2.778 (1.269–6.081) CA: 5.196 (2.723–9.915) AA: 2.048 (0.36–11.479)	0.011 * <0.001 * 0.415
	ME + PDR (VTDR)/NPDR (Non-VTDR)	12 + 11, 36 + 31, 2 + 2/13, 29, 2	CC: 2.041 (0.958–4.352) CA: 6.491 (3.349–12.580) AA: 2.048 (0.365–11.479)	0.065 <0.001 * 0.415
	PDR + NPDR/ME	11 + 13, 31 + 29, 2 + 2/12, 36, 2	CC: 2.375 (1.101–5.122) CA: 3.095 (1.669–5.742) AA: 2.048 (0.365–11.479)	0.027 * <0.001 * 0.415
***IL-18*** ***(−137 G/C)***	ME + NPDR/PDR	9 + 11, 38 + 32, 3 + 2 /5, 36, 2	GG: 4.882 (1.741–13.691) GC: 5.617 (2.874–10.977) CC: 2.590 (0.489–13.724)	0.003 * <0.001 * 0.263
	ME + PDR (VTDR)/NPDR (Non-VTDR)	9 + 5, 38 + 36, 3 + 2 /11, 32, 2	GG: 1.324 (0.565–3.104) GC: 9.250 (4.513–18.958) CC: 2.590 (0.489–13.724)	0.518 <0.001 * 0.263
	PDR + NPDR/ME	5 + 11, 36 + 32, 2 + 2 /9, 38, 3	GG: 1.951 (0.812–4.688) GC: 4.911 (2.551–9.456) CC: 1.349 (0.293–6.213)	0.135 <0.001 * 0.701
***IL-8*** ***(−251 A/T)***	ME + NPDR/PDR	5 + 5, 36 + 33, 9 + 7 /6, 24, 13	AA: 1.752 (0.608–5.050) AT: 9.684 (4.851–19.334) TT: 1.282 (0.576–2.854)	0.299 <0.001 * 0.543
	ME + PDR (VTDR)/NPDR (Non-VTDR)	5 + 6, 36 + 24, 9 + 13 /5, 33, 7	AA: 2.371 (0.788–7.136) AT: 3.571 (1.917–6.656) TT: 3.857 (1.552–9.587)	0.125 <0.001 * 0.004 *
	PDR + NPDR/ME	6 + 5, 24 + 33, 13 + 7 /5, 36, 9	AA: 2.371 (0.788–7.136) AT: 2.656 (1.443–4.887) TT: 2.582 (1.103–6.046)	0.125 0.002 * 0.029 *

Logistic regression; *: Significance (*p* < 0.05); OR: Odds Ratio; 95% CI: 95% Confidence Interval; **CC:** Cytosine-Cytosine; **CA:** Cytosine-Adenine; **AA:** Adenine-Adenine; **TT:** Thymine-Thymine; **AT:** Adenine-Thymine; **GG:** Guanine-Guanine; **GC:** Guanine-Cytosine; Note: ME: Macular Edema, NPDR: Non-Proliferative Diabetic Retinopathy, PDR: Proliferative Diabetic Retinopathy, VTDR: Vision-Threatening Diabetic Retinopathy (defined here as ME + PDR). Subgroups used in combinations are mutually exclusive for that specific statistical model to ensure unique patient counts per analysis.

**Table 7:** Adjusted multivariate risk analysis of IL-18 and IL-8 genotypes.

*Gene Variations*	*Comparison Groups*	*Genotypes*	*AOR (95% CI)*	Adjusted p-Value *
***IL-18*** ***(−607 C/A)***	ME + NPDR/PDR	CC CA AA	1.15 (0.55–2.40) 2.25 (1.35–3.75) 1.05 (0.25–4.45)	0.655 ***0.002*** * 0.945
	ME + PDR (VTDR)/NPDR (Non-VTDR)	CC CA AA	1.05 (0.45–2.45) 2.55 (1.45–4.50) 1.10 (0.20–6.05)	0.915 ***0.001*** * 0.915
	PDR + NPDR/ME	CC CA AA	1.20 (0.50–2.85) 1.95 (1.10–3.45) 1.05 (0.25–4.55)	0.685 0.004 * 0.945
***IL-18*** ***(−137 G/C)***	ME + NPDR/PDR	GG GC CC	1.85 (0.65–4.25) 2.25 (1.35–3.85) 1.25 (0.35–5.45)	0.255 **0.002** * 0.655
	ME + PDR (VTDR)/NPDR (Non-VTDR)	GG GC CC	1.15 (0.45–2.85) 2.85 (1.55–5.25) 1.35 (0.30–6.15)	0.715 ***0.001*** * 0.685
	PDR + NPDR/ME	GG GC CC	1.45 (0.55–3.85) 2.85 (1.55–5.25) 1.35 (0.30–6.15)	0.715 ***0.001*** * 0.685
***IL-8*** ***(−251 A/T)***	ME + NPDR/PDR	AA AT TT	1.15 (0.55–2.45) 2.80 (1.60–4.90) 1.10 (0.45–2.75)	0.755 0.002 * 0.825
	ME + PDR (VTDR)/NPDR (Non-VTDR)	AA AT TT	1.25 (0.55–2.85) 2.85 (1.55–5.25) 1.30 (0.50–3.40)	0.585 0.003 * 0.595
	PDR + NPDR/ME	AA AT TT	1.20 (0.45–3.20) 2.10 (1.25–3.50) 1.15 (0.40–3.35)	0.710 ***0.004*** * 0.795

This analysis adjusted for age, sex, hypertension, CAD, family history, smoking, and alcohol consumption. Significance was determined using the Bonferroni correction (α = 0.005); *: Significance (*p* < 0.05).

## Data Availability

The datasets produced and/or analyzed throughout this current investigation are not available to the public but can be taken from the corresponding author upon a reasonable request.

## Supplementary Materials

The following supporting information can be downloaded at: https://www.mdpi.com/article/10.3390/jcm15135207/s1, Table S1: Allele-Specific PCR protocols for IL-18 variations; Table S2: PCR-RFLP procedure for the IL-8 variation; Table S3: Comparison of clinical and demographic parameters; Table S4: Adjusted multivariate logistic regression analysis for independent risk factors of DR.

## Abbreviations

The following abbreviations are used in this manuscript:

AS-PCR	Allele-Spesifik PCR
DME	Diabetic Maculer Edeme
DR	Diabetic Retinopathy
EDTA	Ethylenediaminetetraacetic acid
eGFR	Estimated Glomerular Filtration Rate
IL-8	Interleukin-8
IL-18	Interleukin-18
NPDR	Non-proliferative Diabetic Retinopathy
PCR	Polymerase Chain Reaction
PDR	Proliferative Diabetic Retinopathy
RFLP	Restriction Fragment Length Polymorphism
VEGF	Vasküler Endotelyal Büyüme Faktörü
VTDR	Vision-Threatening Diabetic Retinopathy
