# Supplementary material for: Genetic Susceptibility to Diabetic Retinopathy in the Thrace Region: Role of IL-18 (−607 C/A, −137 G/C) and IL-8 (−251 A/T) Variations
Source: J Clin Med. 2026 Jul 3;15(13):5207. doi: 10.3390/jcm15135207 (PMC13363530; doi:10.3390/jcm15135207)
Supplement: Supplementary file 1 [file jcm-15-05207-s001.zip › Supplemental Table S1 .pdf]

Supplemental Table S1. Allele-Specific PCR protocols for IL-18 variations.

Gene Variation	*Primer Sequences (5′ - 3′)*	*PCR Conditions*	*Expected Product Lengths*
IL-18 (-607 C/A)	**RP:** 5'-TAACCTCATTCAGGACTTCC-3'<br>**F1P:** 5'-GTTGCAGAAAGTGTAAAAATTATTAC-3'<br>**F2P:** 5'-GTTGCAGAAAGTGTAAAAATTATTAA-3'<br>**FCP:** 5'-CTTTGCTATCATTCCAGGAA-3'	3 minutes at 94°C<br>20 seconds at 94°C<br>20 seconds at 50°C } 40 cycle<br>20 seconds at 72°C<br>5 minutes at 72°C	**CC:** 196bp (F1P)<br>**CA:** 196bp (F1P and F2P)<br>**AA**: 196bp (F2P)
IL-18 (-137 G/C)	**RP:** 5'-AGGAGGGCAAAATGCACTGG-3'<br>**F1P:** 5'-CCCCAACTTTTACGGAAGAAAAG-3'<br>**F2P:** 5'-CCCCAACTTTTACGGAAGAAAAC-3'<br>**FCP:** 5'-CCAATAGGACTGATTATTCCGCA-3'	3 minutes at 94°C<br>20 seconds at 94°C<br>20 seconds at 54°C } 40 cycle<br>20 seconds at 72°C<br>5 minutes at 72°C	**GG:** 261bp (F1P)<br>**GC:** 261bp (F1P and F2P)<br>**CC**: 261bp (F2P)

This table describes the parameters for the IL-18 promoter variations, which do not require restriction enzymes. Allele-Specific PCR Components: 50 ng DNA, forward/reverse primers, 1x PCR buffer, 3mM $MgCl_2$, 1.25 U Taq DNA polymerase. PCR products were observed in 2% agarose gel electrophoresis.

RP: Reverse primer; **F1P:** Forward 1 primer; **F2P:** Forward 2 primer; **FCP:** Forward control primer.
