# Supplementary material for: Genetic Susceptibility to Diabetic Retinopathy in the Thrace Region: Role of IL-18 (−607 C/A, −137 G/C) and IL-8 (−251 A/T) Variations
Source: J Clin Med. 2026 Jul 3;15(13):5207. doi: 10.3390/jcm15135207 (PMC13363530; doi:10.3390/jcm15135207)
Supplement: Supplementary file 1 [file jcm-15-05207-s001.zip › Supplemental Table S2.pdf]

Supplemental Table S2. PCR-RFLP procedure for the IL-8 variation.

Gene Variation	*Primer Sequences (5′ - 3′)*	*PCR Conditions*	*RE*	*Digested Product Lengths*
***IL-8* (-251 A/T)**	**FP:** 5′-CCATCATGATAGCATCTGT-3′<br>**RP:** 5′-CCACAATTTGGTGAATT-3′	5 minutes at 94^0C<br>30 seconds at 94^0C ⎫<br>30 seconds at 56^0C ⎬ 35 cycle<br>1 minutes at 72^0C ⎭<br>8 minutes at 72^0C	VspI (AseI)	**AA:** 152bp, 21bp<br>**AT** 173bp, 152bp, 21bp<br>**TT** 173bp (Undigested)<br>(21bp is not observed)

This table describes the parameters for the IL-8 variation, involving post-PCR enzymatic digestion.

RFLP Components: PCR reaction products, 1x Buffer Tango, dH_2O, 5 Units of EcoRI restriction enzyme.

RFLP products were observed in 2.5% agarose gel electrophoresis
(Note: 19bp fragment is not typically observed).

FP: Forward primer; **RP:** Reverse primer; **RE:** Restriction Enzyme
