# Supplementary material for: Genetic Susceptibility to Diabetic Retinopathy in the Thrace Region: Role of IL-18 (−607 C/A, −137 G/C) and IL-8 (−251 A/T) Variations
Source: J Clin Med. 2026 Jul 3;15(13):5207. doi: 10.3390/jcm15135207 (PMC13363530; doi:10.3390/jcm15135207)
Supplement: Supplementary file 1 [file jcm-15-05207-s001.zip › Supplemental Table S4 Revise.pdf]

Supplemental Table S4. Adjusted multivariate logistic regression analysis for independent risk factors of DR

Clinical and demographic variables	*Adjusted OR (AOR)*	*95% CI*	*Adjusted p-value**
Hypertension (+)	3.95	2.15–7.15	***<0.001****
Familial history of DM (+)	5.20	3.15–10.50	***<0.001****
CAD	4.02	2.10–7.85	***<0.001****
Alcohol (+)	1.35	0.75–2.40	0.280
Smoking (+)	1.42	0.85–2.55	0.120

Adjusted Multivariate Logistic Regression Analysis for Independent Risk Factors of DR
*Bonferroni correction applied (adjusted $\alpha = 0.005$); AOR: Adjusted Odds Ratio; CI: Confidence Interval; (+): Available.
Bold and italic font indicates statistical significance ($p < 0.05$).
CAD: Coronary Artery Disease; DM: Diabetes Mellitus
