# Supplementary material for: Genetic Susceptibility to Diabetic Retinopathy in the Thrace Region: Role of IL-18 (−607 C/A, −137 G/C) and IL-8 (−251 A/T) Variations
Source: J Clin Med. 2026 Jul 3;15(13):5207. doi: 10.3390/jcm15135207 (PMC13363530; doi:10.3390/jcm15135207)
Supplement: Supplementary file 1 [file jcm-15-05207-s001.zip › Supplemental Table S3 Revise.pdf]

Supplemental Table S3. Comparison of clinical and demographic parameters

Clinical and demographic parameters	***Patient group (n=88)***	***Control group (n=88)***	***p***	***OR; 95% Confidence Interval***
Age, mean ± SD	63.625 ± 9.228	63.659 ± 6.523	0.978[a]	
Sex, n (%) Male / Female	48 (54.5%) / 40 (45.5%)	49 (55.7%) / 39 (44.3%)	0.880[b]	
Hypertension (+)	45 (51.1%)	16 (18.2%)	***<0.001***[c*]	4.709; 2.376-9.335
Familial history of DM (+)	37 (42.0%)	9 (10.2%)	***<0.001***[c*]	6.368; 2.836-14.302
CAD	26 (29.5%)	7 (8.0%)	***<0.001***[c*]	4.853; 1.977-11.908
Alcohol (+)	20 (22.7%)	8 (9.1%)	***0.016***[c*]	2.941; 1.218-7.101
Smoking (+)	16 (18.2%)	10 (11.4%)	0.206[c]	1.733; 0.739-4.066

[a]Independent Samples t-test

[b]Chi-squared (χ^2) test

[c]Binary Logistic Regression analysis

(+): Available. Bold and italic font indicates statistical significance ($p < 0.05$).

CAD: Coronary Artery Disease; DM: Diabetes Mellitus; SD: Standard Deviation.
